# The convergent application of metabolites from *Avena sativa* and gut microbiota to ameliorate non-alcoholic fatty liver disease: a network pharmacology study

**DOI:** 10.1186/s12967-023-04122-6

**Published:** 2023-04-17

**Authors:** Ki-Kwang Oh, Sang-Jun Yoon, Su-Been Lee, Sang Youn Lee, Haripriya Gupta, Raja Ganesan, Satya Priya Sharma, Sung-Min Won, Jin-Ju Jeong, Dong Joon Kim, Ki-Tae Suk

**Affiliations:** Institute for Liver and Digestive Diseases, College of Medicine, Hallym University, Chuncheon, 24252 Korea

**Keywords:** Non-alcoholic fatty liver disease, Secondary metabolites, Gut microbiota, *Avena sativa*, VEGFA, PI3K-Akt signaling pathway

## Abstract

**Background:**

Non-alcoholic fatty liver disease (NAFLD) is a serious public health issue globally, currently, the treatment of NAFLD lies still in the labyrinth. In the inchoate stage, the combinatorial application of food regimen and favorable gut microbiota (GM) are considered as an alternative therapeutic. Accordingly, we integrated secondary metabolites (SMs) from GM and *Avena sativa* (AS) known as potent dietary grain to identify the combinatorial efficacy through network pharmacology.

**Methods:**

We browsed the SMs of AS via Natural Product Activity & Species Source (NPASS) database and SMs of GM were retrieved by gutMGene database. Then, specific intersecting targets were identified from targets related to SMs of AS and GM. The final targets were selected on NAFLD-related targets, which was considered as crucial targets. The protein–protein interaction (PPI) networks and bubble chart analysis to identify a hub target and a key signaling pathway were conducted, respectively. In parallel, we analyzed the relationship of GM or AS─a key signaling pathway─targets─SMs (GASTM) by merging the five components via RPackage. We identified key SMs on a key signaling pathway via molecular docking assay (MDA). Finally, the identified key SMs were verified the physicochemical properties and toxicity in silico platform.

**Results:**

The final 16 targets were regarded as critical proteins against NAFLD, and Vascular Endothelial Growth Factor A (VEGFA) was a key target in PPI network analysis. The PI3K-Akt signaling pathway was the uppermost mechanism associated with VEGFA as an antagonistic mode. GASTM networks represented 122 nodes (60 GM, AS, PI3K-Akt signaling pathway, 4 targets, and 56 SMs) and 154 edges. The VEGFA-myricetin, or quercetin, GSK3B-myricetin, IL2-diosgenin complexes formed the most stable conformation, the three ligands were derived from GM. Conversely, NR4A1-vestitol formed stable conformation with the highest affinity, and the vestitol was obtained from AS. The given four SMs were no hurdles to develop into drugs devoid of its toxicity.

**Conclusion:**

In conclusion, we show that combinatorial application of AS and GM might be exerted to the potent synergistic effects against NAFLD, dampening PI3K-Akt signaling pathway. This work provides the importance of dietary strategy and beneficial GM on NAFLD, a data mining basis for further explicating the SMs and pharmacological mechanisms of combinatorial application (AS and GM) against NAFLD.

**Supplementary Information:**

The online version contains supplementary material available at 10.1186/s12967-023-04122-6.

## Background

Non-alcoholic fatty liver disease (NAFLD) is characterized by excessive fat accumulation in liver tissues, especially, in liver parenchyma [1]. Its pathophysiological spectrum is encompassed in simple fatty liver (SFL) to non-alcoholic steatohepatitis (NASH), eventually reaching at liver cirrhosis and hepatocellular carcinoma (HCC), via proceeding liver fibrosis (LF) [2]. Also, NAFLD is associated with diverse metabolic disorders: obesity, diabetes mellitus, hypertension, and cardiovascular diseases [3]. The surging incidence and complicated etiology cause clinical burden, searching for effective therapeutic strategies in epidemiological, and behavior approach of NAFLD patients with a primary option [4]. Recently, although several therapeutic options have been reported to ameliorate NAFLD, its noticeable therapeutic preferences are yet to be determined [5]. Formalized treatments for NAFLD are not documented and an available option is to be counselled concerning healthy lifestyle: regimen, abstinence of high fats and carbohydrates, and frequent physical exercise [6–8].

In the incomplete project, we pioneered the secondary metabolites (SMs) from *Avena sativa* (AS; known as oat) and gut microbiota (GM) to identify the key SMs in both AS and GM for the treatment of NAFLD. Furthermore, AS has a wide spectrum of pharmacological activities such as antioxidant, anti-inflammatory, antidiabetic and anticholesterolemic efficacy [9]. The AS is an ancient grain utilized as an important grain from primitive times, suggesting that AS can diminish cholesterol, control satiety, and even make positive effects on gastrointestinal (GI) health [10, 11]. Currently, several studies have demonstrated that natural products can regulate body metabolism including anti-obesity and anti-diabetes [12].

Specially, the flavonoids from AS ameliorated hyperlipidemia caused by high-fat-diet via modulating bile acid and GM in mice [13]. The AS supplement has alleviating effects to lower the blood pressure in hypertensive groups by increasing *Bifidobacterium* and *Spirillum* [14]. Furthermore, the bioactives of AS are key players to regulate the beneficial GM to relieve metabolic disorders: obesity, atherosclerosis, and even osteoporosis [15]. Thereby, it elicits that AS is a significant modulator to control GM community.

In parallel, GM in human intestine is significant community to control the physiological responses for host [16]. Some favorable GM (known as probiotics) can convert into key SMs (known as postbiotics) is implicated in many metabolic disorders including NAFLD [17]. Furthermore, probiotics and postbiotics are vital effectors to regulate PI3K/AKT pathway by interconnecting with AMP-activated kinase (AMPK) pathway [18, 19]. Some reports to approve the effects of postbiotics on inflammatory pathways have been shown different experimental results due to different postbiotic mixture in media and its derivative structures [20]. Collectively, our study is to manifest key SMs from AS and GM to keep consistent results for the treatment of NAFLD. With the help of network pharmacology concept, we performed integrative analysis to pinpoint crucial elements: key GM, signaling pathway(s), target(s), and SMs. Network pharmacology (NP) is a systemic methodology to decipher the complex biological pathways, which is a valuable tool to elucidate efficacy of complex natural products [21]. NP has been developed as a new methodology in drug discovery as it combines scattered valuable information with data science [22]. As a matter of fact, NP decodes the complicated interaction between compounds, targets, and diseases from holistic viewpoint on multiple components [23]. Most recently, a merged GM and NP study was contributed to decode the roles of GM against diarrhea-predominant irritable bowel syndrome (IBS-D), indicating that eighteen GM with treatment of Chinese traditional medicine were critical components to alleviate IBS-D [24]. Furthermore, key SMs of alcoholic liver diseases (ALD) and NAFLD were deciphered with the NP analysis [25, 26].

**Fig. 1:**
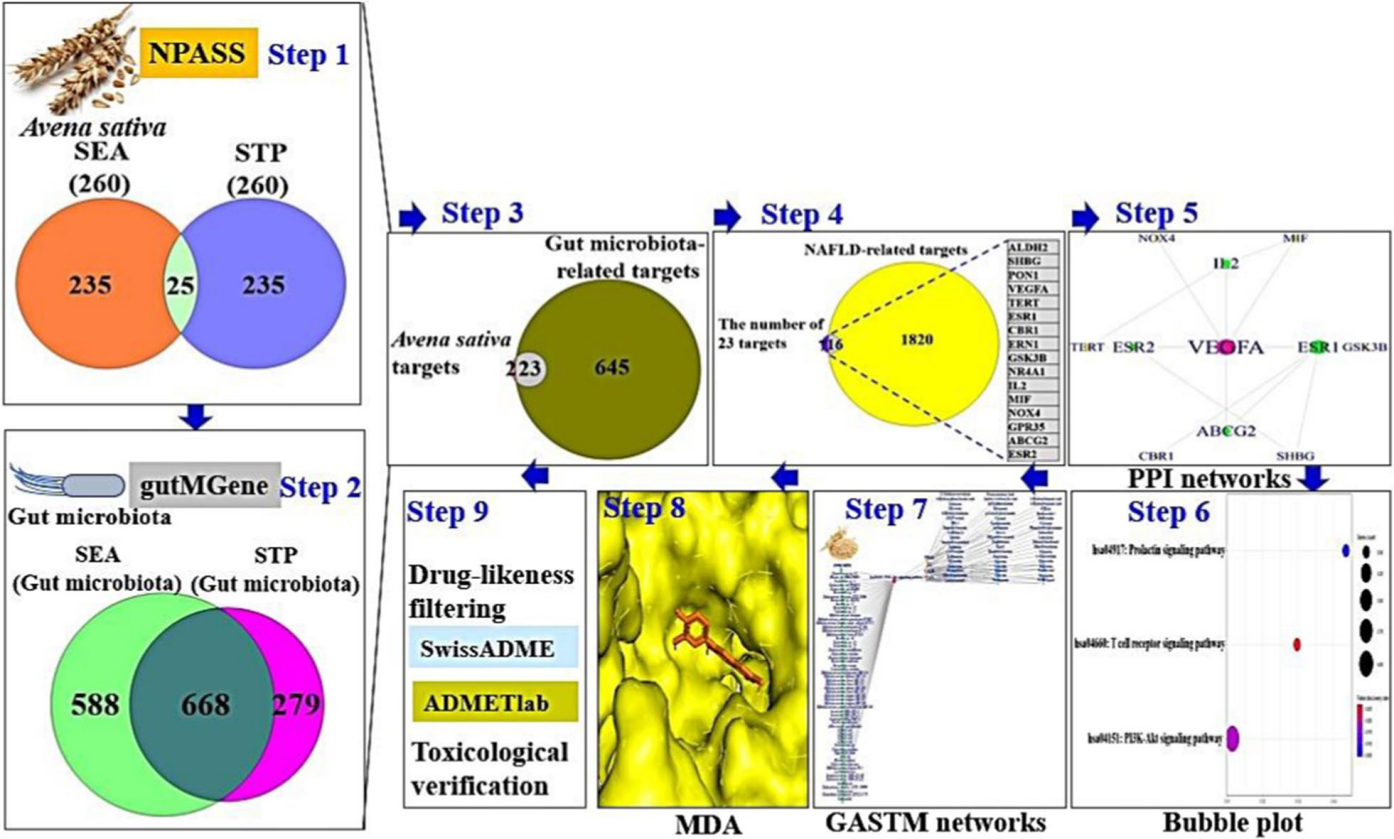
The workflow of this study

It is believed that NP might be a key to decrypt the therapeutic issue in dilemma, ending up with combinatorial application. As aforementioned, our study has established that the combinatorial application of AS and GM is to be expected as an alternative therapeutic strategy for NAFLD. Thus, this approach might be given critical hints to further clinical trials and advancement of the combined applications with AS and GM. The process of this study is displayed in Fig. 1. Given the limited microbiome data, the pharmacological pathway of combining AS and GM in the alleviation of NAFLD is only dependent upon mining data. The integrative methodology to reveal the combinatorial effects on AS and GM provides significant clues for favorable diet and well-designed microbiota compositions. The purpose of this study was to uncover combinatorial effects on both AS and GM for the treatment on NAFLD.

## Materials and methods

### The employment of the web-based public bioinformatics

At present, the development of data science provides a great deal of information related to biological pathways between compounds and targets, which can be a platform by merging the important databases. Based on it, we gathered valuable data to utilize NP as a drug discovery methodology. We profiled the available bioinformatics resources in Table 1.

**Table 1:** The lists of accessible databases for the study

No	Databases	Short description	URL
1	ADMETlab 2.0	Cheminformatics to identify physicochemical properties or compound toxicities	https://admetmesh.scbdd.com/
2	DisGeNET	Bioinformatics of target-gene relationships on human	https://www.disgenet.org/
3	gutMGene	Microbiome database to identify metabolites of gut microbiota	http://bio-annotation.cn/gutmgene
4	NPASS	Database of natural herbal plants	http://bidd.group/NPASS/
5	Online Mendelian Inheritance in Man (OMIM)	Human database to identify between targets and diseases	https://www.omim.org/
6	Pro Tox-II	Cheminformatics to predict compound toxicities	https://tox-new.charite.de/protox_II/index.php?site=home
7	Similarity Ensemble Approach (SEA)	Cheminformatics to decode targets on compounds	https://sea.bkslab.org/
8	String	Bioinformatics to identify protein–protein interaction networks	https://string-db.org/
9	SwissADME	Cheminformatics to predict the drug-like properties	http://www.swissadme.ch/
10	SwissTargetPrediction (STP)	Cheminformatics to survey targets on small compounds	http://www.swisstargetprediction.ch/
11	VENNY 2.1	Venn diagram drawing tool to compare each list of constituents	https://bioinfogp.cnb.csic.es/tools/venny/

### The identification of SMs and its targets from AS

Natural Product Activity & Species Source (NPASS) database (http://bidd.group/NPASS/) (accessed on 28 September 2022) was utilized to select the significant SMs from AS [27], indicating that targets related to the SMs were retrieved by Similarity Ensemble Approach (SEA) (https://sea.bkslab.org/) (accessed on 28 September 2022) [28] and SwissTargetPrediction (STP) (http://www.swisstargetprediction.ch/) (accessed on 28 September 2022) [29]. With the exactness and rigor, the intersecting targets between SEA and STP were considered as important targets associated with SMs from AS. It was defined as AS-related targets. Crucially, SEA database is a mining platform to select some major targets linked to targets, developed by Dr Shoichet’s group. It is to be specified that the number of 23 in 30 targets extracted by SEA was confirmed by experimentation [30]. Apparently, STP has been used to identify the putative targets for ligands, for instance, the attained targets for cudraflavone C hit the mark experimentally [31].

### The selection of SMs and its targets from GM

The gutMGene database was used to obtain the SMs converted by GM (http://bio-annotation.cn/gutmgene/) (accessed on 29 September 2022) [32]. The obtained SMs were input into both SEA and STP platform to select the targets. The overlapping targets identified by SEA and STP were identified as critical targets associated with SMs from GM. It is defined as GM-related targets.

### The determination of core targets from AS and GM against NAFLD

The intersecting targets were identified between AS-related targets and GM-related targets, which were considered as significant targets for combinatorial therapeutics. The NAFLD- related targets extracted by DisGeNET (https://www.disgenet.org/) (accessed on 30 September 2022) [33] and OMIM (https://www.omim.org/) (accessed on 30 September 2022) [34]. Finally, we selected the core targets against NAFLD, by comparing the targets between combinatorial therapeutics’ targets and NAFLD-related targets.

### The protein–protein interaction networks

We utilized String database (https://string-db.org/) (accessed on 01 October 2022) [35] to identify protein–protein interaction (PPI) networks, which was described by R Package. On the PPI networks, we found a target with the highest degree value, thus it was to be defined as a key target to ameliorate NAFLD.

### The construction of bubble plot

The construction of bubble plot was established by Kyoto Encyclopedia of Gene and Genomes (KEGG) pathway enrichment analysis. The signaling pathways on the bubble plot were depicted, according to Rich factor value. We discerned a key signaling pathway for the treatment of NAFLD, suggesting that the mechanism might be inhibitive effect on NAFLD. The bubble plot was constructed by R package.

### The construction of GM or AS—a key signaling pathway-targets-SMs (GASTM) networks

We described GASTM network to know the relationships of each component: GM or AS, a key signaling pathway, targets, and secondary metabolites. The GASTM network was constructed by utilizing R Package. Taken together with GM or AS, a key signaling pathway, targets, and SMs as nodes, matching associations above components were assembled with Microsoft Excel, then input into R package to identify the interaction network of GASTM against NAFLD.

### Molecular docking assay (MDA)

The Molecular docking assay (MDA) was implemented with AutodockTools-1.5.6 to understand what the most significant SMs in both GM and AS are. Commonly, the threshold of AutodockTools-1.5.6. was fitted as -6.0 kcal/mol [36] or SM with lowest Gibbs energy (the greatest negative value) was regarded as the uppermost SM to have therapeutic value in the treatment of NAFLD. The SMs were selected as.sdf format from PubChem (https://pubchem.ncbi.nlm.nih.gov/) (accessed on 02 October 2022), changing into.pdb format via Pymol tool. The.pdb format was transformed into.pdbqt format to prepare for the MDA on targets. The targets were selected by the Protein Data Bank (PDB) (https://www.rcsb.org/) (accessed on 01 October 2022) for.pdb format, which were switched into.pdbqt format by setting parameter in AutodockTools-1.5.6. The MDA was conducted on.pdbqt format by preparing for conformer between SMs and targets. The docking site was set in cubic box (x = 40 Å, y = 40 Å, and z = 40 Å) in a central point of each target.

### The validation of drug-likeness and toxic parameters on the uppermost SMs

The properties of drug-likeness on the uppermost SMs were performed by SwissADME (http://www.swissadme.ch/) (accessed on 02 October 2022) [37]. The filtering standard was based on Lipinski’s rule: Molecular weight (< 500 g/mol) or Topological Polar Surface Area (TPSA) (< 140 Å^2^) or Moriguchi octanol–water partition coefficient (MLogP) (≤ 4.15) or Hydrogen Bonding Acceptor (HBA) (< 10) or Hydrogen Bonding Donor (HBD) (≤ 5). To accept the rule, the molecules should not be violated more than 2 parameters out of 5 parameters. The toxicity of the uppermost SMs was confirmed by ADMETlab 2.0 [38] and ProTox-II [39], its parameters are as follows: Human ether-a-go-go-related gene (hERG) [40]; Human Hepatotoxicity (H-HT) [41]; Carcinogens [42]; Cytotoxicity [43]; and Eye corrosion [44].

## Results

### The secondary metabolites (SMs) of AS and its targets

**Fig. 2:**
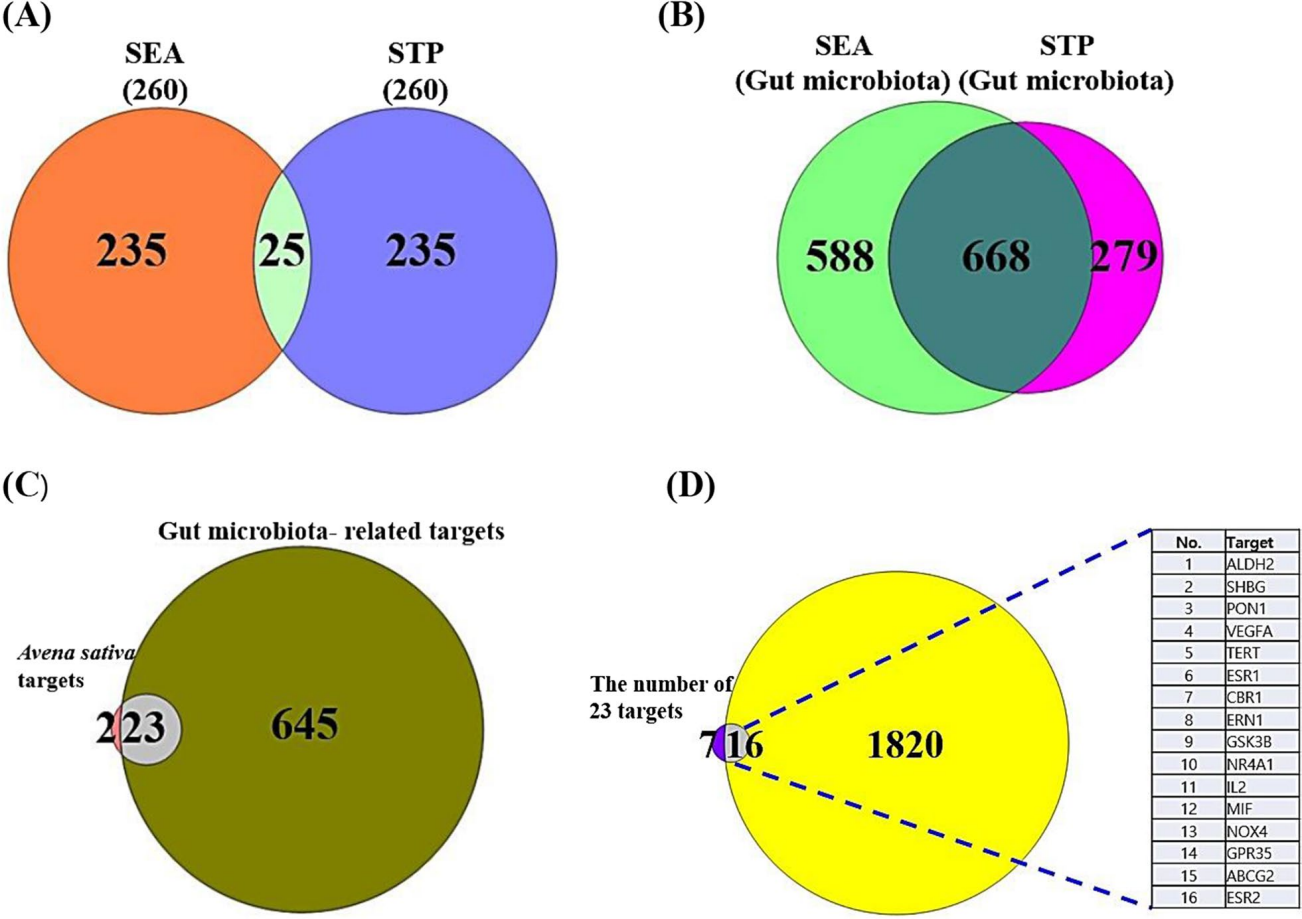
**A** The number of overlapping 25 targets from *Avena sativa* between SEA and STP. **B** The number of overlapping 668 targets from gut microbiota between SEA and STP. **C** The number of overlapping 23 targets between *Avena sativa* and gut microbiota. **D** The number of overlapping 16 targets via intersecting targets of *Avena sativa* and gut microbiota against NAFLD

The number of 12 SMs from AS was documented from NPASS database, all of which were accepted by Lipinski’s rule (Table 2). The targets related to the 12 SMs were retrieved by SEA (260) and STP (260), thus, the number of 25 overlapping targets was identified between the cheminformatics databases (Fig. 2A). The 25 overlapping targets were considered as significant protein-coding gene associated with AS.

**Table 2:** The physicochemical properties of the secondary metabolites (SMs) of *Avena sativa* (AS) identified by NPASS

No	Compounds	PubChem ID	Lipinski's Rules				Lipinski's Violations	Bioavailability Score	TPSA(Å^2^)
			MW	HBA	HBD	MLog P			
			< 500	< 10	≤ 5	≤ 4.15	≤ 1	> 0.1	< 140
1	Castanin	5281704	298.29	5	1	1.01	0	0.55	68.90
2	4',7,8-Trihydroxyisoflavone	5466139	270.24	5	3	0.52	0	0.55	90.90
3	(-)-Epicatechin	72276	290.27	6	5	0.24	0	0.55	110.38
4	(-)-Catechin	73160	290.27	6	5	0.24	0	0.55	110.38
5	Protocatechuic Acid	72	154.12	4	3	0.40	0	0.55	77.76
6	Spermidine	1102	145.25	3	3	0.08	0	0.55	64.07
7	Formononetin	5280378	268.26	4	1	1.33	0	0.55	59.67
8	(3S)-Vestitol	177149	272.30	4	2	1.87	0	0.55	58.92
9	Raspberryketone	21648	164.20	2	1	1.74	0	0.55	37.30
10	Medicarpin	336327	270.28	4	1	1.87	0	0.55	47.92
11	Vestitol	92503	272.30	4	2	1.87	0	0.55	58.92
12	Mdl-26752	492218	244.42	4	4	1.03	0	0.55	76.10

### The secondary metabolites (SMs) of GM

We identified the number of 208 SMs (Additional file 1: Table S1) in gutMGene database, the targets connected to the 208 SMs were confirmed by SEA (1256) and STP (947) (Additional file 1: Table S1). The overlapping 668 targets were regarded as significant protein-coding genes related to SMs from GM (Additional file 1: Table S1) (Fig. 2B).

### The overlapping targets between AS-related targets and GM-related targets

The number of 23 targets was identified between the number of 25 overlapping targets from AS and 668 targets from GM, suggesting that the 23 targets are significant targets to exert the combinatorial efficacy on both AS and GM (Fig. 2C).

### The identification of core targets against NAFLD

The number of 23 targets obtained from the combined AS-related targets and GM-related targets was compared with NAFLD-associated targets (1836) (Additional file 1**: **Table S1), the final 16 targets were identified as bona fide targets to be expected to exert combinatorial efficacy on AS-based and GM-based application (Fig. 2D**)**.

### A key target on PPI network and MDA

**Fig. 3:**
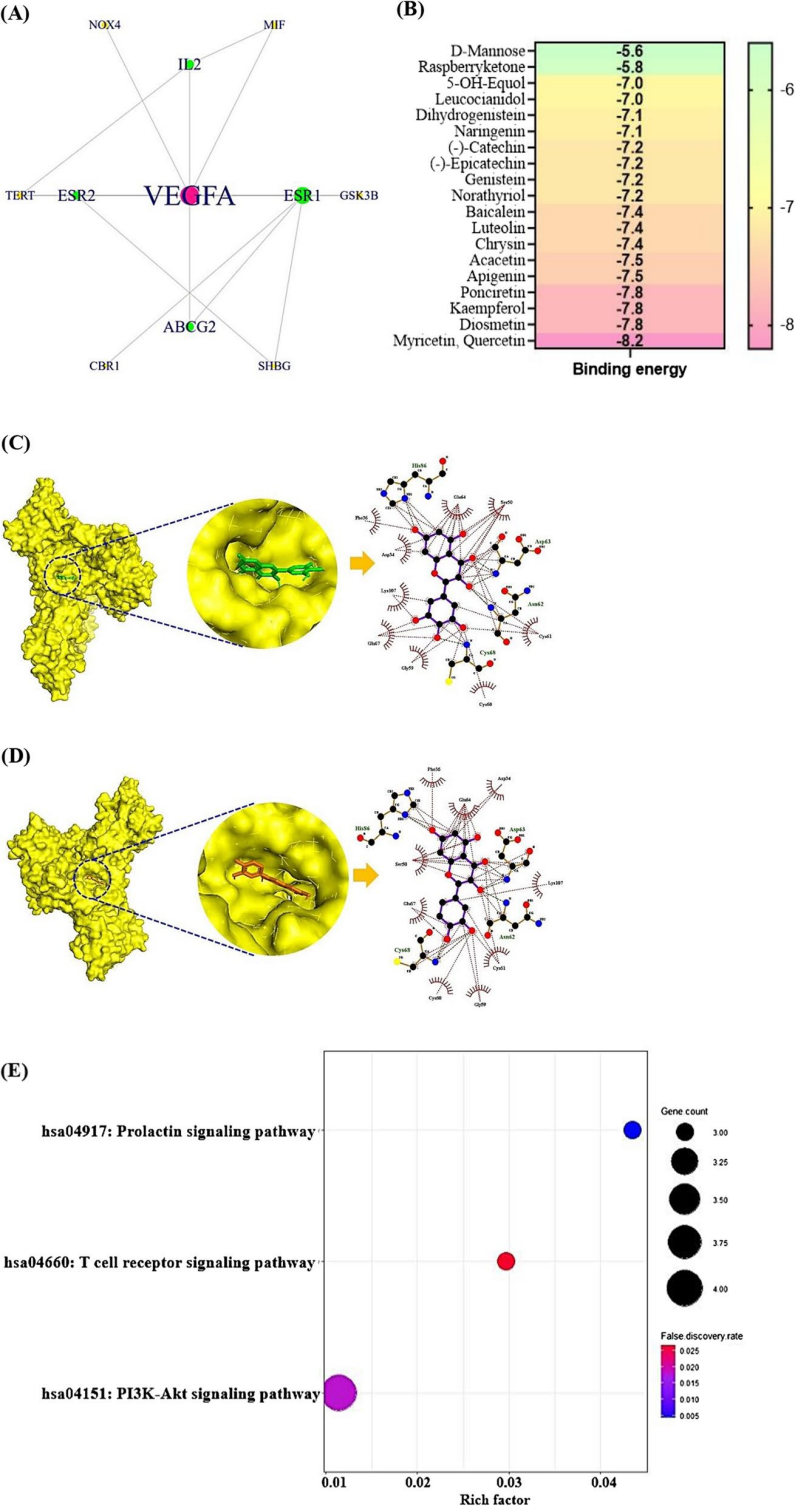
**A** PPI networks. **B** A heatmap of binding energy on 20 SMs against VEGFA. **C** The conformer of myricetin-VEGFA. **D** The conformer of quercetin-VEGFA. **E** Bubble plot of 3 signaling pathways associated with occurrence and development of NAFLD

The final 16 targets PPI network consisted of 11 nodes and 17 edges, and 5 (ALDH2, PON1, ERN1, NR4A1, and GPR35) out of 16 targets were not interacted with one another **(**Fig. 3A). The Vascular Endothelial Growth Factor A (VEGFA) in the networks was the highest degree of value, followed by ESR1 (7), ESR2 (3), IL2 (3), and TERT (3) (Table 3). We considered the VEGFA as the uppermost target against NAFLD. In addition, results of the MDA showed that the binding energy of myricetin, quercetin was -8.2 kcal/mol as the lowest score in the number of 20 SMs (Fig. 3B), indicating that these SMs (myricetin, quercetin) could exert a potent binding affinity with VEGFA (Fig. 3C, D). The GM is enabled to convert myricitrin into myricetin, the applicable GM is *Escherichia sp. 12*, *Escherichia sp. 33*, and *Enterococcus sp. 45* [45]. Moreover, quercitrin by *Bacteroides sp. 45* [46], rutin by *Bifidobacterium dentium*, *Bacteroides uniformis,* and *Bacteroides ovatus* [47, 48]*,* avicularin *Bacillus sp. 46* [49], myricitrin by *Enterococcus sp. 45*, and *Escherichia sp. 33* [45], isoquercitrin by *Enterococcus casseliflavus* [50], can convert into quercetin. It implies that the GM is beneficial probiotics to produce favorable postbiotics, in parallel, myricetin and quercetin are good effectors to bind stably on VEGFA.

**Table 3:** The degree value of targets on PPI network

No	Target name	Degree of value
1	VEGFA	8
2	ESR1	7
3	ESR2	3
4	IL2	3
5	TERT	3
6	ABCG2	2
7	GSK3B	2
8	MIF	2
9	SHBG	2
10	CBR1	1
11	NOX4	1

### Bubble plot and GASTM network

The bubble plot provided by PPI shows three signaling pathways: Prolactin signaling pathway, T cell receptor signaling pathway, and PI3K-Akt signaling pathway (Fig. 3E). Among the three signaling pathways, PI3K-Akt signaling pathway linked directly to VEGFA was considered as a key signaling pathway (Table 4). In parallel, VEGFA, IL2, GSK3B, and NR4A1 related to PI3K-Akt signaling pathway are regarded as promising targets significantly. Noticeably, PI3K-Akt signaling pathway had the lowest rich factor, suggesting that the signaling pathway might function as antagonistic mode. It means that lower rich factor can be defined as less number of expressed genes in annotated signaling pathways [51].

**Table 4:** The description of signaling pathways related to NAFLD in the study

KEGG ID & description	Target	False discovery rate
hsa04917: prolactin signaling pathway	ESR1, ESR2, GSK3B	0.0134
hsa04660: T cell receptor signaling pathway	FYN, IL2, GSK3B	0.0267
hsa04151: PI3K-Akt signaling pathway	VEGFA, IL2, GSK3B, NR4A1	0.0450

**Fig. 4:**
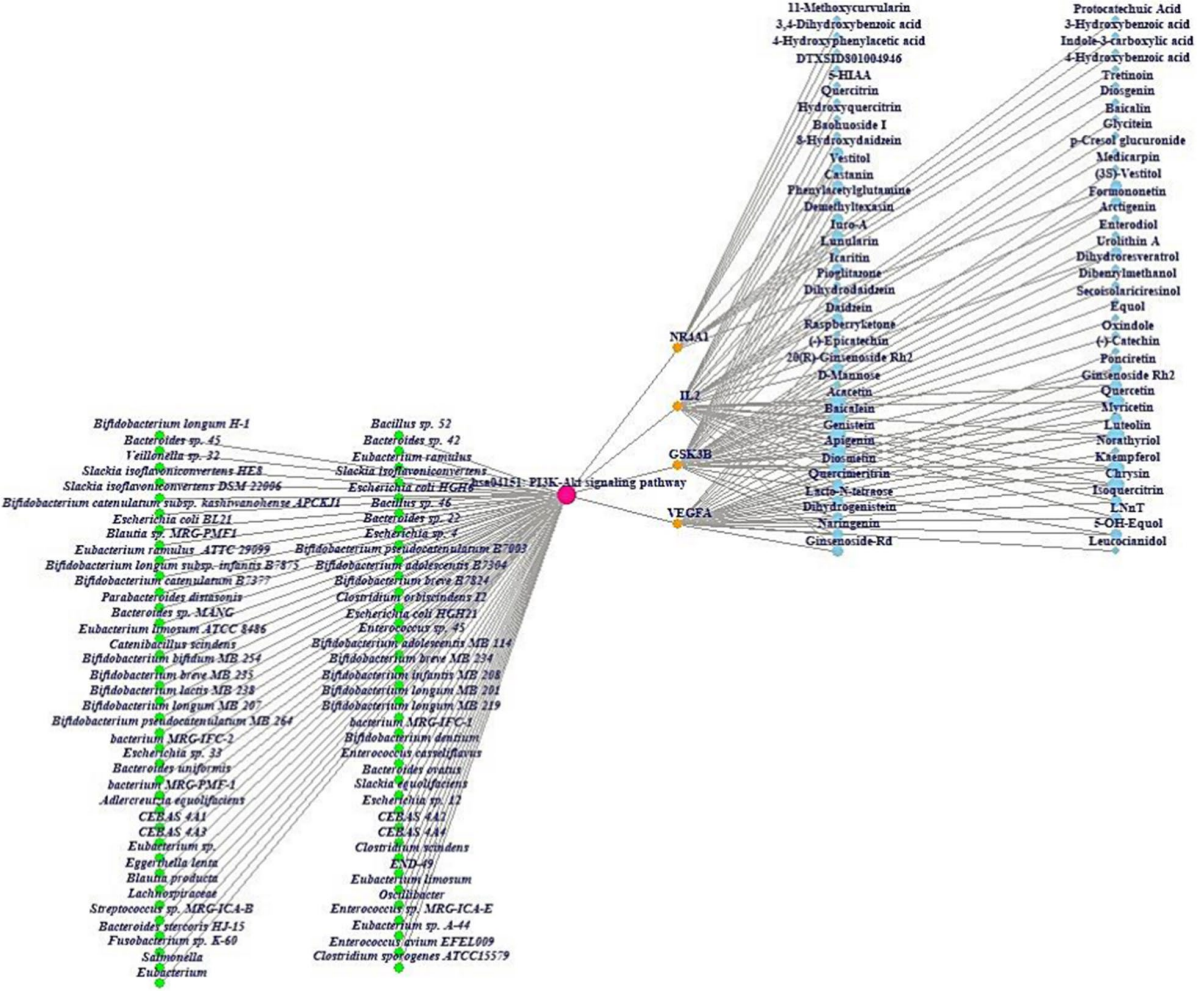
The GM or AS- a key signaling pathway-targets-SMs (GASTM) network (122 nodes and 155 edges)

The GASTM network shows the relationships between GM (60 nodes, green circle) or AS (1 node*,* green circle), PI3K-Akt signaling pathway (1 node, green circle), targets (4 nodes, orange circle), and metabolites (56 nodes, blue sky circle), consisting of 122 nodes and 155 edges (Fig. 4). On a holistic viewpoint, the integrated four components can exert therapeutic effects, orchestrate with each other against NAFLD.

### Molecular docking assay

**Fig. 5:**
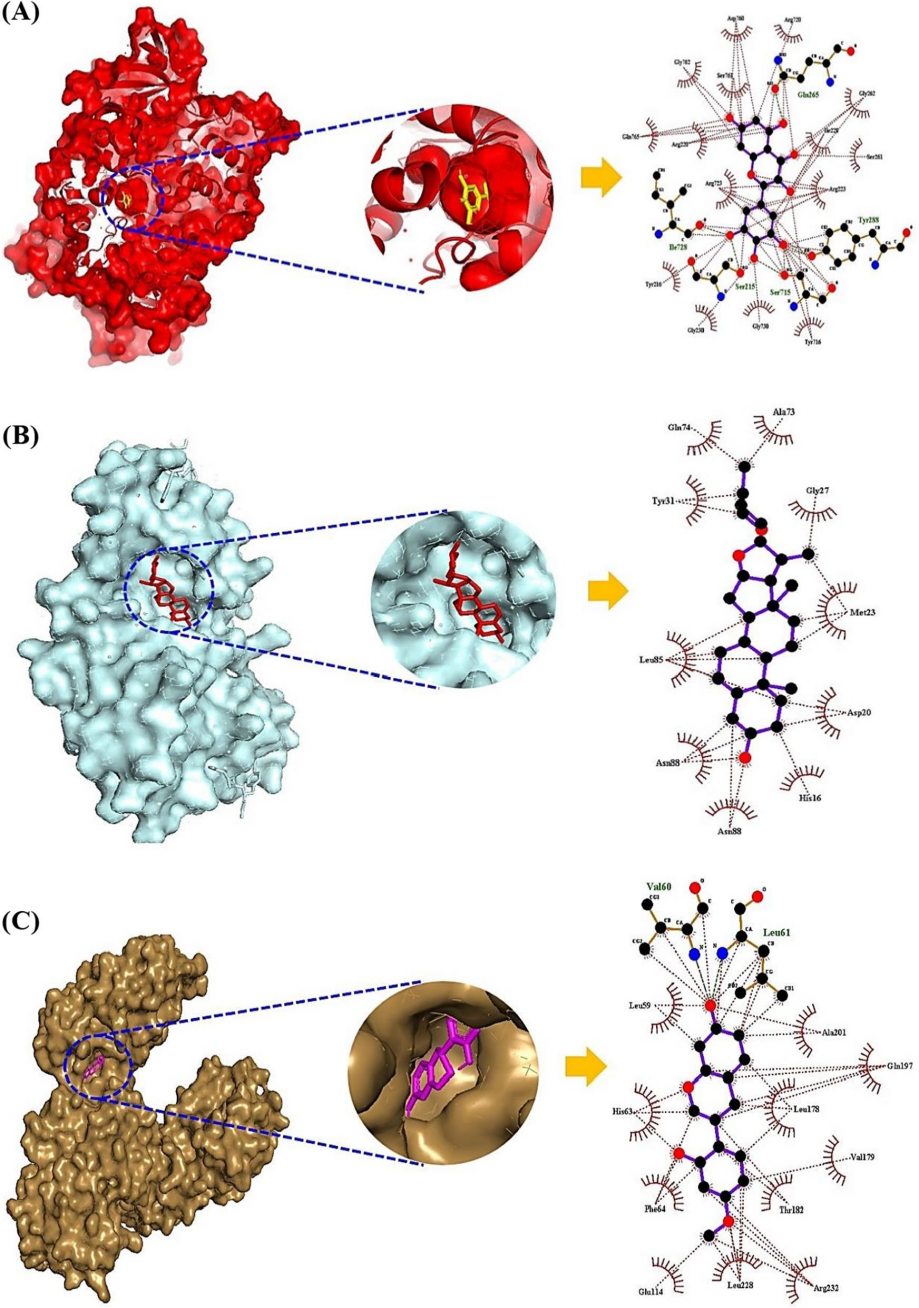
The results of molecular docking assay (MDA). **A** myricetin-GSK3B. **B** diosgenin-IL2. **C** vestitol-NR4A1

The molecular docking assay (MDA) shows what the promising SM(s) are on the PI3K- Akt signaling pathway, and the key SM(s) are derived from either GM or AS. As mentioned previously, the key SMs (myricetin, and quercetin) of VEGFA were derived from GM (*Escherichia sp. 12*, *Escherichia sp. 33*, *Enterococcus sp. 45*, *Bacteroides sp. 45, Bifidobacterium dentium*, *Bacteroides uniformis, Bacteroides ovatus*, *Bacillus sp. 46,* and *Enterococcus casseliflavus*). Likewise, myricetin on GSK3B had the highest affinity with -10.6 kcal/mol (Fig. 5A), originated from *Escherichia sp. 12*, *Escherichia sp. 33*, and *Enterococcus sp. 45*. Diosgenin bound most stably to IL2 had the greatest affinity with -9.1 kcal/mol (Fig. 5B), which can be converted from SCHEMBL20481776 (PubChem ID: 135312912) [52]. However, GM can convert diosgenin are yet to be revealed. Vestitol on NR4A1 formed the most stable conformer with -9.0 kcal/mol (Fig. 5C), the vestitol was derived from AS. The information of the MDA was profiled in Additional file 2: Table S2. Collectively, the combinatorial application of AS and beneficial GM can involve in the treatment on NAFLD via the PI3K-Akt signaling pathway by multiple-compounds, and multiple-targets.

### The verification of drug-likeness and toxicity on key SMs

**Fig. 6:**
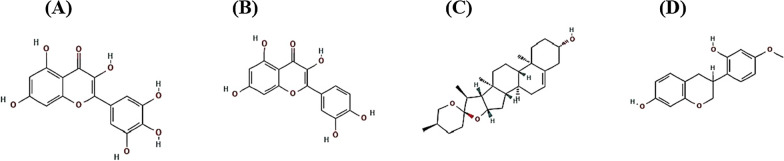
The chemical structures of four key SMs. **A** myricetin. **B** quercetin. **C** diosgenin. **D** vestitol

The number of four SMs (myricetin, quercetin, diosgenin, and vestitol) was accepted by Lipinski’s rule, thus, which could be important agents to develop therapeutics. Accordingly, the parameters of toxicity were all confirmed: hERG, Human Hepatotoxicity, Carcinogens, Cytotoxicity, and Eye corrosion. Thus, the identified four SMs are promising candidates against NAFLD (Table 5). The four SMs had no physicochemical hindrances to be therapeutic agents. The chemical structures of the key SMs were exhibited in Fig. 6.

**Table 5:** The verification of drug-likeness and toxicity on key SMs

Parameters	Secondary metabolite			
	Myricetin	Quercetin	Diosgenin	Vestitol
Hydrogen bonding acceptor (HBA); < 10	8	7	3	4
Hydrogen bonding donor (HBD); ≤ 5	6	5	1	2
Moriguchi octanol–water partition coefficient (MLog P); ≤ 4.15	− 1.08	− 0.56	4.94	1.87
Topological polar surface area (TPSA(Å^2^)) < 140	151.59	131.36	38.69	58.92
Lipinski’s rule ≤ 1	1	0	1	0
hERG (hERG blockers)	Non-inhibitor	Non-inhibitor	Non-inhibitor	Non-inhibitor
Human hepatotoxicity (H-HT)	Negative	Negative	Negative	Negative
Carcinogens	Non-carcinogens	Non-carcinogens	Non-carcinogens	Non-carcinogens
Cytotoxicity	Inactive	Inactive	Inactive	Inactive
Eye corrosion	Negative	Negative	Negative	Negative

## Discussion

The PPI network shows that VEGFA is the uppermost target to regulate other 10 targets. The key SMs (myricetin, quercetin) have been revealed by MDA, which could form the most stable conformers on VEGFA. The myricetin diminishes the lipid synthesis in liver cell and inflammatory response by tuning GM [53]. Additionally, an animal test demonstrated that the quercetin enhances NAFLD by alleviating inflammation, free-radicals, and lipid degradation in type 2 diabetic mice [54]. Commonly, expression level of the VEGFA increases during obesity, and neutralization of VEGFA relieves the metabolic disorders occurred by diet [55]. It elicits that VEGFA inhibitors can be candidates to improve lipid metabolic dysfunction including NAFLD.

The three signaling pathways confirmed by PPI are related to occurrence and development of NAFLD, which concisely discussed in Table 6.

**Table 6:** The description of the three signaling pathways related to occurrence and development of NAFLD

Signaling pathway	Target or metabolism	Activation or inhibition	Effect	Notes
Prolactin signaling pathway	Prolactin	Activation	NAFLD ↓	-Prolactin is an endogenous polypeptide with approximately 23 kda, which has negative relationships concerning NAFLD [66] -Prolactin receptor expression is diminished in obese subjects under NAFLD, the downregulation of which exacerbates NAFLD [66] -Thus, it implies that activation of prolactin signaling pathway can be a therapeutic strategy against NAFLD
T cell receptor signaling pathway	Complex (MHC) class I (CD8 +)	Inhibition	NAFLD ↑	-In NAFLD, reduction of CD8 + diminished the liver inflammation and led to hepatic stellate cell (HSC) inactivation [67]
	Complex (MHC) class II (CD4 +)	Inhibition	NAFLD ↓	-The dysfunction of lipid metabolism in NAFLD subjects (human and mouse) caused the reduction of CD4 + in liver [68, 69] -It has been implicated that CD4 + T cells decrease in the development of NAFLD while CD8 + T cells escalate in progression of HCC initiated by NAFLD [68, 70]
PI3K-Akt signaling pathway	The synthesis of free fatty acids (FFAs) in organs	Inhibition	NAFLD ↓	-In obese subjects, over-circulating of FFAs driven by PI3K-Akt signaling pathway can influence on negative side effects to organs, resulting in imbalance of glucose and lipid metabolism [71]
	The synthesis of triglyceride in hepatocytes	Inhibition	NAFLD ↓	-The up-regulation of PI3K-Akt signaling pathway accelerates the synthesis of triglyceride in hepatocytes [72]

↓: improvement; ↑: deterioration

The most stable SM on IL2 was diosgenin, suggesting that the diosgenin is a representative compound in saponin derivatives [56]. A report shows that diosgenin reduces triglyceride content in the liver, and stimulates the excretion of cholesterol [57]. Another report to support the therapeutic efficacy of diosgenin demonstrated that diosgenin interrupts the lipid absorption in intestine, triggers cholesterol transformation into bile acid and its elimination as well as interferes with lipid biosynthesis [58]. Also, IL2 inhibitor is a potent therapeutic agent to treat diverse inflammatory responses including NAFLD [59, 60]. It elicits that diosgenin is not only an effector to control lipid content but can also be used as NAFLD alleviator. A typical SM conformed to Glycogen Synthase Kinase 3 Beta (GSK3B) was myricerin with highly therapeutic values such as antioxidant, antidiabetic, antiinflammation, and even anticancer [61]. Noticeably, GSK3 antagonist alleviates hepatic steatosis which is accompanied by mitochondrial abnormality [62]. A report demonstrated that GSK3B inhibitor can be a promising therapeutic effector to control NAFLD [63]. Thus, it has been supported that antagonists of GSK3B might be significant agents for the treatment of NAFLD. A representative SM bound to Nuclear receptor subfamily 4 group A member 1 (NR4A1) was vestitol derived from AS, which is a species of isoflavonoid [64]. The vestitol is known as a potent anti-inflammatory agent by reducing leukocyte rolling [64]. At present, few studies of vestitol have been reported. The NR4A1 involved in chronic inflammatory state and dysfunction of lipid metabolism in type2 diabetic (T2D) patients [65]. It implies that the inhibition of NR4A1 can help regulate lipid biosynthesis against NAFLD.

Overall, this study shows that dampening of PI3K-Akt signaling pathway might be a potential mechanism to relieve NAFLD. In detail, the key effectors that we suggested are myricetin, quercetin from *Escherichia sp. 12*, *Escherichia sp. 33*, *Enterococcus sp. 45*, *Bacteroides sp. 45, Bifidobacterium dentium*, *Bacteroides uniformis, Bacteroides ovatus*, *Bacillus sp. 46,* and *Enterococcus casseliflavus*. However, the GM that converts diosgenin is still veiled. Although there were no evident relationships either positive or negative feedback between AS and nine GM, we could expect the high possibility as relievers on NAFLD, obtaining the four SMs: (1) myricetin, (2) quercetin, (3) diosgenin from GM, and (4) vestitol from AS (known as oatmeal).

**Fig. 7:**
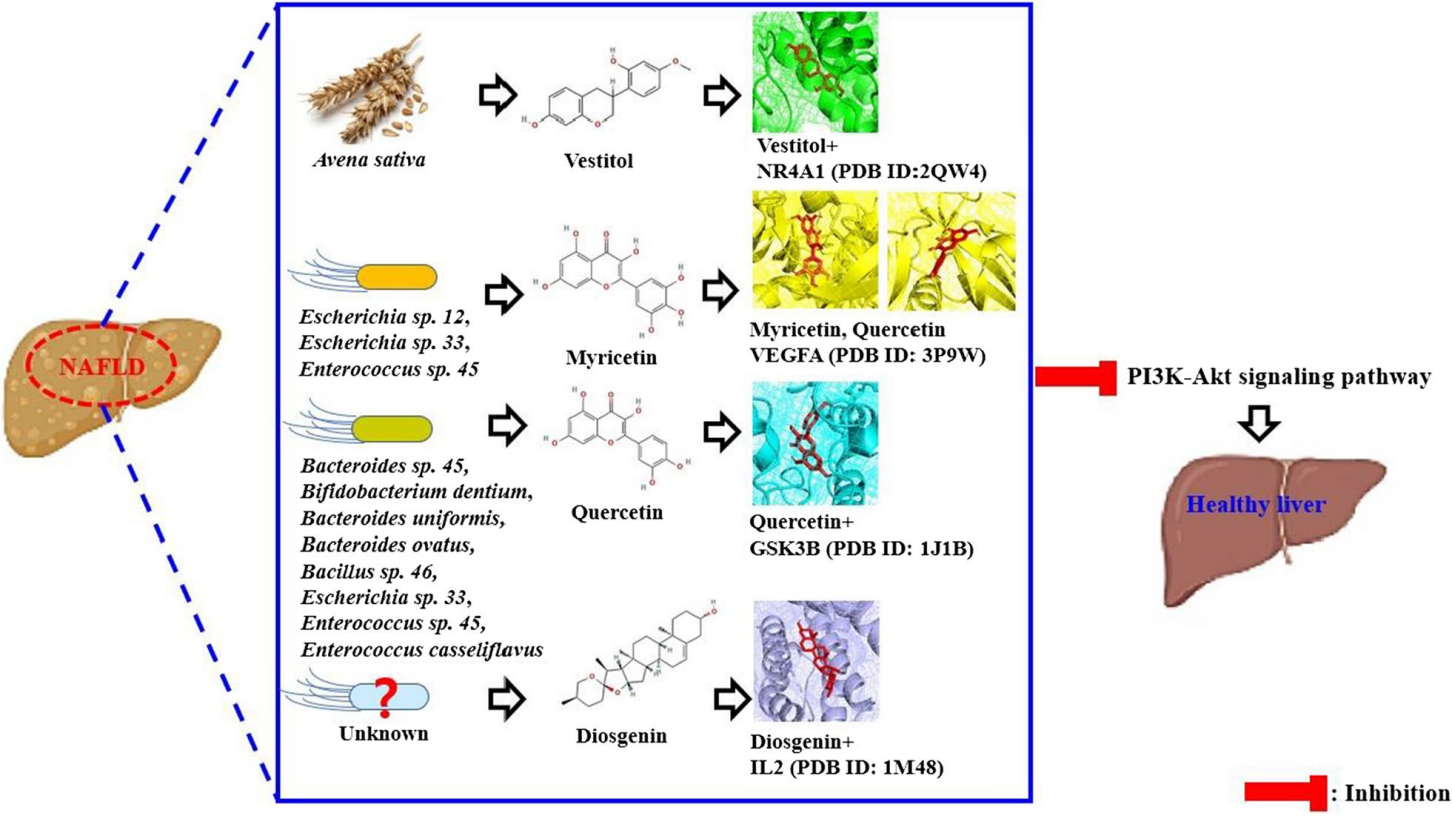
The key findings in this study

All in all, these findings shed light on the importance of GM as therapeutics, and AS with auxiliary role in the context NAFLD. Despite that, our study is required to do clinical tests and extensive investigation with rigorousness. The key findings of this study are represented in Fig. 7.

### The pros and cons of this study

From the incorporation of NP, this study exploratory organizes on the key GM, signaling pathways, targets, and SMs with the application of AS in NAFLD treatment, suggesting theoretical evidence for further clinical verification. As limited borderline of NP, the pharmacological pathway of integrating analysis on AS or GM against NAFLD is only dependent on data-driven analysis, and its combinatorial effects, the interaction between AS and GM in vivo were ignored, which is integral to validate the authenticity through preclinical and clinical tests. Hence, the performance of our analysis needs to be advanced more, for instance, by integrating new dataset continually. At this point, our approach platform is easy to merge new data to improve the performance and might be a hallmark to elucidate the relationships between diet and GM. In addition, our study provides a rationale for how to improve accuracy prior to clinical trials.

## Conclusion

In conclusion, our study highlights the therapeutic effects and mechanisms of the treatment on NAFLD via combinatorial application: gut microbiota (GM), and *Avena sativa* (AS), indicating antagonists (myricetin, quercetin, diosgenin, and vestitol) to inhibit PI3K-Akt signaling pathway. These findings provide a new insight to utilize the endogenous species (gut microbiota) and exogenous species (*Avena sativa*) on microbiome-based therapeutics. However, this study should be taken in vitro or in vivo experimentation into consideration to uncover bona fide pharmacological efficacy.

## Supplementary Information

**Additional file 1: Table S1**: The number of 208 SMs of gut microbiota from gutMGene; The number of 1256 targets from SEA; The number of 947 targets from STP; The number of 668 targets between SEA (1256) and STP (947); The number 1836 targets related to NAFLD.

**Additional file 2: Table S2**: The binding energy and coordinated amino acid bonds of SMs on each key target.

## Data Availability

All data generated or analyzed during this study are included in this published article (and its Additional files).

## Abbreviations

AMPK: AMP-activated Kinase
AS: Avena sativa
GASTM: GM or AS- a key Signaling pathway-Targets-SMs
GM: Gut Microbiota
HBA: Hydrogen Bonding Acceptor
HBD: Hydrogen Bonding Donor
hERG: Human Ether-a-go-go-Related Gene
H-HT: Human HepatoToxicity
KEGG: Kyoto Encyclopedia of Gene and Genomes
LF: Liver Fibrosis
MDA: Molecular Docking Assay
MLogP: Moriguchi octanol–water partition coefficient
NAFLD: Non-Alcoholic Fatty Liver Disease
NASH: Non-Alcoholic SteatoHepatitis
NP: Network Pharmacology
NPASS: Natural Product Activity & Species Source
PDB: Protein Data Bank
PPI: Protein–Protein Interaction
SEA: Similarity Ensemble Approach
SM: Secondary Metabolite
SMs: Secondary Metabolites
STP: SwissTargetPrediction
T2D: Type2 Diabetic
TPSA: Topological Polar Surface Area
VEGFA: Vascular Endothelial Growth Factor A
